# Inhibition of Respiratory RNA Viruses by a Composition of Ionophoric Polyphenols with Metal Ions

**DOI:** 10.3390/ph15030377

**Published:** 2022-03-20

**Authors:** Topaz Kreiser, Dor Zaguri, Shreya Sachdeva, Rachel Zamostiano, Josef Mograbi, Daniel Segal, Eran Bacharach, Ehud Gazit

**Affiliations:** 1 The Shmunis School of Biomedicine and Cancer Research, Tel Aviv University, Tel Aviv 6997801, Israel; topazkreiser@mail.tau.ac.il (T.K.); dorzaguri@mail.tau.ac.il (D.Z.); shreya@mail.tau.ac.il (S.S.); r.zamostiano@gmail.com (R.Z.); dsegal@tauex.tau.ac.il (D.S.); 2 Independent Researcher, Tel Aviv 6937940, Israel; yossi@vit4pro.com; 3 Sagol Interdisciplinary School of Neuroscience, Tel Aviv University, Tel Aviv 6997801, Israel; 4 BLAVATNIK CENTER for Drug Discovery, Tel Aviv University, Tel Aviv 6997801, Israel

**Keywords:** zinc, polyphenols, flavonoids, antiviral, respiratory RNA viruses, replication inhibition

## Abstract

Controlling the infectivity of respiratory RNA viruses is critical, especially during the current SARS-CoV-2 pandemic. There is an unmet need for therapeutic agents that can reduce viral replication, preferably independent of the accumulation of viral mutations. Zinc ions have an apparent activity as modulators of intracellular viral RNA replication and thus, appear attractive in reducing viral RNA load and infectivity. However, the intracellular concentration of zinc is usually too low for achieving an optimal inhibitory effect. Various herbal polyphenols serve as excellent zinc ionophores with known antiviral properties. Here, we combined zinc picolinate with a collection of flavonoids, representing commonly used polyphenols. Copper was added to avoid ionic imbalance during treatment and to improve efficacy. Each component separately, as well as their combinations, did not interfere with the viability of cultured A549, H1299, or Vero cells in vitro as determined by MTT assay. The safe combinations were further evaluated to determine antiviral activity. Fluorescence-activated cell sorting and quantitative polymerase chain reaction were used to evaluate antiviral activity of the combinations. They revealed a remarkable (50–95%) decrease, in genome replication levels of a diverse group of respiratory RNA viruses, including the human coronavirus OC43 (HCoV-OC43; a betacoronavirus that causes the common cold), influenza A virus (IAV, strain A/Puerto Rico/8/34 H1N1), and human metapneumovirus (hMPV). Collectively, our results offer an orally bioavailable therapeutic approach that is non-toxic, naturally sourced, applicable to numerous RNA viruses, and potentially insensitive to new mutations and variants.

## 1. Introduction

Respiratory virus infections cause a wide range of symptoms and illnesses, resulting in substantial morbidity and mortality worldwide, as seen in the coronavirus disease 2019 (COVID-19) pandemic [1]. This disease, caused by a novel coronavirus—the severe acute respiratory syndrome coronavirus 2 (SARS-CoV-2), has spread globally during the past two years at an unprecedented rate [2]. The virus is highly contagious, spreads easily among people, has a variable incubation period, and leads to the development of respiratory disease. The infection is known to affect older people more severely. Additional risk factors for severe disease include various chronic comorbidities such as obesity, hypertension, and diabetes [3]. While some patients infected by SARS-CoV-2 exhibit no symptoms, others present a spectrum of symptoms including fever, dry cough, muscle weakness, and some may experience pneumonia [4,5,6], comparable to the manifestation of flu [7]. Recent evidence suggests that severely ill patients of respiratory diseases tend to have a high concentration of pro-inflammatory cytokines [8,9], and an increasing number of studies indicate that a “cytokine storm” may contribute to the danger of mortality from COVID-19 [10]. According to the World Health Organization, as of 6 March 2022, over 433 million COVID-19 cases and consequently, more than 5.9 million deaths have been reported globally, with an estimated death rate of 1.4%. This makes COVID-19 the second pandemic of the twenty-first century [11].

The vaccines against SARS-CoV-2, authorized by the US Food and Drug Administration (FDA), are based on the virus envelope spike protein [12,13] and effectively prevent severe illness. However, reaching the target vaccination levels required for herd immunity may take considerable time, especially with new COVID variants [14]. Along with vaccines, the scientific community and Big Pharma have invested much effort in finding antiviral therapeutic agents to either mitigate or halt the spread of SARS-CoV-2 [15]. These attempts have mainly focused on developing neutralizing monoclonal antibodies that target the spike protein [16,17]. However, most of these agents must be administered by infusion in a clinical setting, limiting their potential for widespread use [18]. Importantly, SARS-CoV-2 is evolving rapidly, with new lineages being continuously discovered around the world. The Omicron variant, for example, is characterized by the presence of about 30 mutations in the spike protein. Recent studies have shown that Omicron escapes most therapeutic monoclonal antibodies and, to a large extent, vaccine-elicited antibodies [19,20]. Therefore, even though many people have already been infected with, or vaccinated against, SARS-CoV-2 globally, these data highlight the possibility of reinfection with antigenically different variants, which could mean that current spike-based vaccinations and treatments may turn less effective. Thus, it is probable that the SARS-CoV-2 vaccines would need to be frequently reformulated to match the circulating strains, as is done for seasonal influenza vaccines [21]. Much effort has been invested in identifying spike variants; however, mutations unrelated to the spike protein seem also to contribute to viral adaptation. For example, the emergence of SARS-CoV-2 variants that suppress innate immune responses through changes in the nucleocapsid, Orf9b, and Orf6 proteins suggests that the virus has evolved to allow for more efficient human-to-human transmission [22]. COVID-19 has proven to be difficult to control compared to previous viral outbreaks, and the global response to the pandemic included complete lockdowns, social distancing measures, and population screening policies [11]. This caused an unusual and unexpected impact on several other respiratory diseases. Some being temporarily eradicated, while others have progressed and are resurfacing even during the off-season. Additionally, various respiratory viruses, including influenza virus, adenovirus, rhinovirus/enterovirus, and parainfluenza virus, were co-detected together with SARS-CoV-2 [23,24,25]. Therefore, alternative treatments that halt the spread and morbidity of COVID-19 and other respiratory viral infections are still urgently needed [26,27]. Overall, the SARS-CoV-2 expands the list of other RNA viruses that cause significant morbidity and mortality in human populations worldwide [28,29].

Since several medicinal plants and herbal formulations have proven beneficial for treating viral infections, increasing attention is being paid to natural products for effective treatment toward the COVID-19 pandemic [15]. Here, we describe combinations of natural compounds that are readily available dietary supplements, considered as Generally Recognized as Safe (GRAS) in the US, that exhibit a remarkable synergistic in vitro antiviral activity when combined. The active composition we developed is based on the combination of metal ions with ionophoric polyphenols. Zinc is an essential element required for the function of over 300 enzymes and around 1000 transcription factors. Zinc deficiency or suboptimal levels of zinc cause a range of ailments including severe respiratory infections [30,31,32,33,34]. Zinc demonstrates antiviral activity against a variety of viruses by numerous mechanisms. As reviewed by Read et al. [34], it has been shown that among other mechanisms, zinc inhibits the RNA-dependent RNA polymerase of coronavirus, viral polyprotein cleavage in Rhinovirus, Encephalomyocarditis virus, and foot and mouth disease virus, and it reduces the viral titer and plaque count of the respiratory syncytial virus (RSV).

Indeed, low-dose zinc supplementation in combination with a low amount of zinc ionophores inhibits the RNA-dependent RNA polymerase of various viruses [30,35]. Copper ions, also have a broad antiviral effect, including against coronaviruses [36,37].

Polyphenols, the largest class of bioactive chemicals found in nature, are known for their antiviral activity [38,39]. Flavonoids, the most common group of polyphenolic compounds in the human diet, are found ubiquitously in fruits and vegetables, and studies support their potential use to treat various human diseases [40,41]. Additionally, flavonoids are considered to have notable beneficial health effects, including antibacterial, anti-inflammatory, antioxidant, and a wide range of antiviral activities against several human viruses [15,39,41,42]. Hence, flavonoids are key components of various dietary supplements for the relief of medical conditions.

Based on these considerations, we combined here non-toxic dietary supplements composed of zinc picolinate, copper sulfate, and the flavonoids Epigallocatechin-3-gallate (EGCG), Quercetin, Taxifolin (dihydroquercetin), and Naringenin, that act as zinc ionophores and transport zinc cations through the cell membrane [26,40,42,43,44,45,46,47]. The combination effectively inhibited in vitro viral replication in several cell types, including human lung cells. Importantly, the combination exhibited higher efficacy than its individual components, demonstrating an additive effect. The tested RNA viruses included three respiratory pathogens: the H1N1 subtype influenza A virus (IAV; strain A/Puerto Rico/8/34, also known as PR8 strain) [48] and the human metapneumovirus (hMPV) [49]. Additionally, as a model for SARS-CoV-2, we used HCoV-OC43 [21,50] as it is also a betacoronavirus that targets the human respiratory system [51]. Such an inexpensive combination of dietary supplements would be highly advantageous to have, alongside vaccines, as a safe prevention method affecting various RNA respiratory viruses.

## 2. Results and Discussion

### 2.1. Biocompatibility of the Ingredients and Their Active Combinations

**Figure 1:**
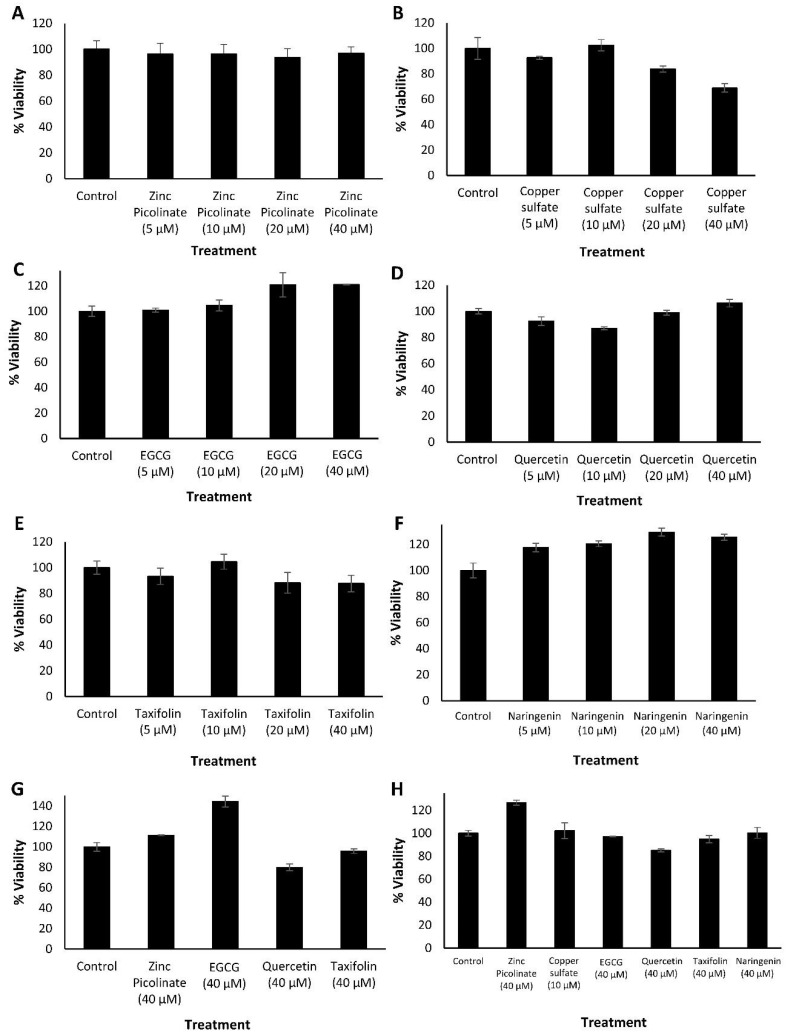
Cytotoxicity evaluation of the examined compounds by MTT. (**A**–**F**). A549 cells treated with media containing the indicated concentrations of (**A**). Zinc picolinate dissolved in phosphate-buffered saline (PBS), (**B**). Copper sulfate dissolved in ultra-pure water (UPW), (**C**). EGCG dissolved in cell culture medium, (**D**). Quercetin dissolved in dimethylsulfoxide (DMSO), (**E**). Taxifolin dissolved in DMSO, (**F**). Naringenin dissolved in DMSO (**G**). H1299 cells and (**H**). Vero cells, treated with the indicated compounds and concentrations. Control cells were incubated with media containing the relevant solvents without the compounds. The cells were incubated with or without the compounds for 24 h, followed by the addition of the MTT reagent. Absorbance was determined at 570 and 680 nm. Results represent three biological repeats; values are means ± SD.

**Figure 2:**
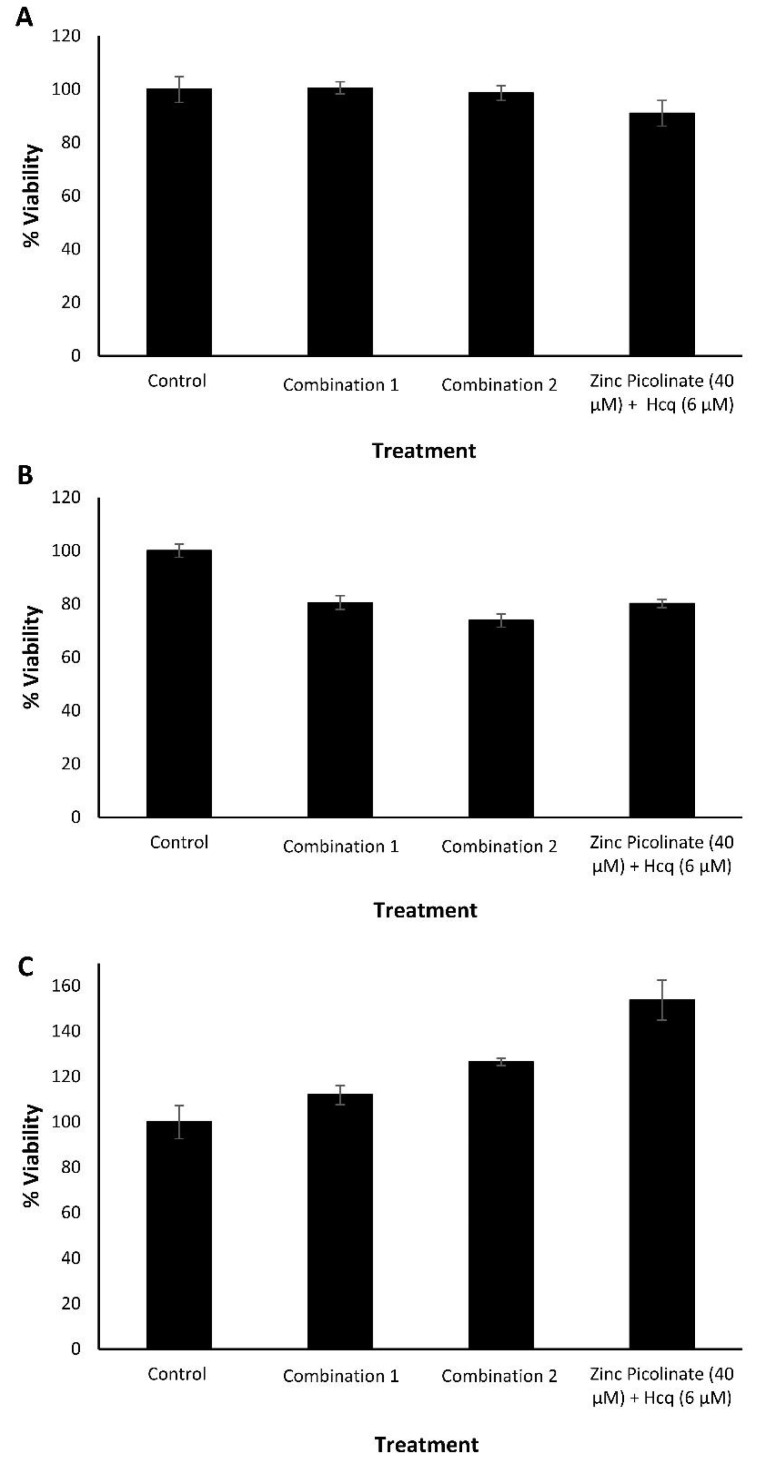
Cytotoxicity evaluation of the natural compound combinations by MTT. (**A**). A549, (**B**). H1299, and (**C**). Vero cells, treated with culture media containing two dietary supplements combinations, or zinc picolinate (40 µM) combined with Hcq (6 µM). Combination 1—zinc picolinate (40 µM), copper sulfate (10 µM), EGCG (40 µM), quercetin (40 µM), and taxifolin (40 µM). Combination 2—zinc picolinate (40 µM), copper sulfate (10 µM), EGCG (40 µM), quercetin (40 µM), taxifolin (40 µM), and naringenin (40 µM). Cells, incubated with a medium containing the relevant solvents in which the compounds were dissolved, served as the control. The cells were incubated with the combinations for 24 h, followed by the addition of the MTT reagent. Absorbance was determined at 570 and 680 nm. The results represent three biological repeats; values are means ± SD.

As a proxy for the safety of the ingredients of the combinations, we tested each compound for its cytotoxicity at several concentrations, as well as of two representative combinations. Cytotoxicity was evaluated using an MTT cell viability assay. Human epithelial carcinoma lung cells (A549), human non-small cell lung carcinoma cells (H1299), African green monkey kidney cells (Vero), and human neuroblastoma cells (SH-SY5Y) were incubated with the indicated compounds for 24 h. Control cells were incubated with a medium containing the cognate compound’s solvents. Figure 1 summarizes the results where viability percentage is normalized to the control that was set as 100% viability. The tested compounds did not impair the viability of either A549 cells (Figure 1A–E), H1299 cells (Figure 1F), Vero cells (Figure 1G), and SH-SY5Y cells (Figure S1A) upon treatment with the indicated selected compounds and concentrations. The results verified that the selected compounds did not possess a significant cytotoxic effect as determined by the MTT assay. Next, we examined the viability of A549, H1299, and Vero cells, incubated with two selected combinations of the compounds. Combination 1 was composed of zinc picolinate (40 µM), copper sulfate (10 µM), EGCG (40 µM), quercetin (40 µM), and taxifolin (40 µM). Combination 2 was composed of zinc picolinate (40 µM), copper sulfate (10 µM), EGCG (40 µM), quercetin (40 µM), taxifolin (40 µM), and naringenin (40 µM). The commercially available medication, hydroxychloroquine (Hcq; 6 µM) together with zinc picolinate (40 µM), was used for comparison since Hcq serves as zinc ionophore and was proposed as an antiviral agent for the treatment of COVID-19. The FDA issued an Emergency Use Authorization (EUA) for Hcq; however, this EUA was later canceled [52,53]. We used a concentration of 6 µM for Hcq as it is two times the equivalent of Hcq drug serum concentration that COVID-19 patients had received [54]. The negative control consisted of cells incubated with medium-containing solvents, but without the tested compounds. The cells were incubated with the abovementioned combinations for 24 h, after which cell viability was assessed. As shown in Figure 2, the combinations did not substantially reduce cell viability in any cell type. Similar results were obtained in SH-SY5Y cells (Figure S1B). Given that the ingredients of these combinations are GRAS dietary supplements, these results suggest that the two tested combinations may be safe for use.

### 2.2. Inhibition of Intracellular Viral Replication by Natural Compound Combinations

#### 2.2.1. Flow Cytometry Analysis and Quantification

**Figure 3:**
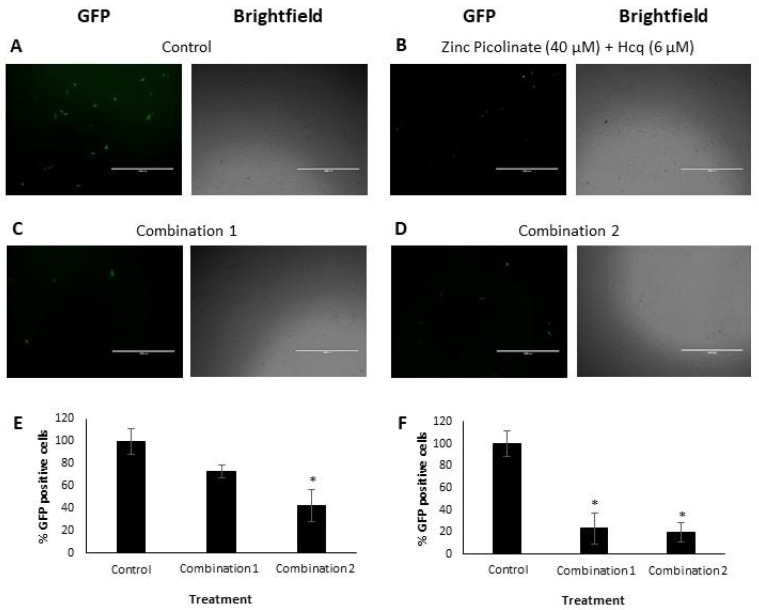
Visualization and quantification of hMPV and IAV replication in Vero and A549 cells, respectively. (**A**–**D**). Representative fluorescence and brightfield images of Vero cells, infected with hMPV and treated with (**A**). Solvents (control), (**B**). Zinc picolinate (40 µM) with Hcq (6 µM), (**C**). Combination 1 and (**D**). Combination 2. Scale bars: 1 mm. (**E**,**F**). FACS analyses. Graphs show the percentages of GFP-positive cells of (**E**). hMPV-infected Vero cells and (**F**). IAV-infected A549 cells for each indicated treatment. Multiplicity of infection (MOI) was 0.6 (hMPV) or 0.75 (IAV). The results represent three biological repeats. Values are means ± SD, Student’s *t*-test, * *p* < 0.05, all statistics are in relation to control.

We next determined the efficacy of the combinations in inhibiting viral replication. A549 lung cells and Vero cells were infected with IAV and hMPV, respectively (Figure 3). The viruses were labeled with mNeonGreen (IAV) and Green Fluorescent Protein (GFP; hMPV). The cells were grown to 90% confluency one day before infection. Four hours before infection, the cells were treated with the abovementioned natural compound combinations: Combination 1 or Combination 2. The combination of zinc picolinate (40 µM) and Hcq (6 µM) was used as a positive control treatment. Cells incubated with a medium containing the cognate solvents, but without the compounds served as a negative control. The treated cells were infected with the IAV for 24 h or with hMPV for 48 h, after which the cells were visualized using fluorescence microscopy. Figure 3A–D shows representative fluorescence and brightfield images of Vero cells infected with hMPV. GFP-positive cells represent virus-infected cells, in which viral replication had occurred [55]. Qualitatively, incubation with the two combinations reduced the number of GFP-positive cells compared to the controls. Infected and treated cells were analyzed using Fluorescence-Activated Cell Sorting (FACS) to quantify this effect. Figure 3E presents the quantification of hMPV infection by displaying the percentage of GFP-positive cells. Samples that were treated with either one of the two combinations exhibited lower percentages of GFP-positive cells, with Combination 2 demonstrating significant inhibition of viral infection. We also demonstrated the inhibitory effect of each of the two combinations for IAV-infected A549 cells (Figure S2 and Figure 3F).

#### 2.2.2. Quantitative Real-Time PCR Analysis

**Figure 4:**
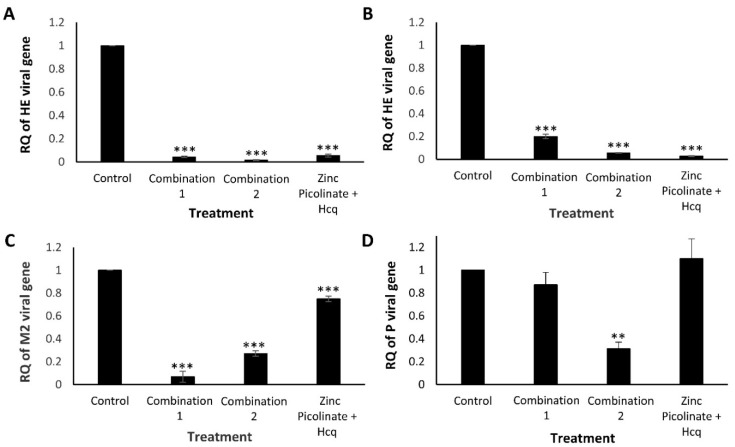
Quantification of HCoV-OC43, hMPV, and IAV replication using qRT-PCR. Cells were treated with control medium, Combination 1, Combination 2, or zinc and Hcq. After four hours, the treated cells were infected, incubated for 24 to 48 h, and total RNA was extracted from the cells. Viral replication was quantitatively assessed by calculating the RQ of the indicated viral genes. RQ of (**A**). HCoV-OC43 HE gene in A549 cells, (**B**). HCoV-OC43 HE gene in H1299 cells, (**C**). IAV M2 gene in A549 cells, and (**D**). hMPV P gene in Vero cells. The results represent three biological repeats. The MOI was 0.03 (HCoV-OC43), 0.6 (hMPV), or 0.75 (IAV). Values are means ± SD, Student’s *t*-test, ** *p* < 0.01, *** *p* < 0.001, all statistics are in relation to control.

To complement and substantiate the abovementioned results, we examined the efficacy of the combinations in inhibiting viral replication using Real-Time quantitative Reverse Transcription PCR (qRT-PCR); this analysis was also extended to HCoV-OC43 and H1299 cells (Figure 4). As described above, the cells were grown to 90% confluency, and four hours before infection were supplemented with the tested combinations. The treated cells were infected with IAV or HCoV-OC43 for an additional 24 or 48 h when infected with hMPV. Next, RNA was purified from the cell lysates, converted to complementary DNA (cDNA), and qRT-PCR was carried out using virus-specific primers to amplify viral genes and quantify viral replication. A Relative Quantity (RQ) index was calculated based on the number of amplification cycles needed to exceed a required threshold normalized to the housekeeping gene Glyceraldehyde-3-phosphate dehydrogenase (GAPDH). The RQ values were also normalized to infected, untreated control cells. The RQ of the viral genes in these cells was normalized to a value of “1”. As can be seen in Figure 4A,B, the levels of the HCoV-OC43 Hemagglutinin-Esterase (HE) gene were reduced significantly when A549 (Figure 4A) or H1299 (Figure 4B) cells were treated with Combination 1 or Combination 2. Figure 4C demonstrates reduced levels of the IAV M2 gene in A549 cells treated with either one of the two combinations, compared to cells treated with zinc and Hcq. Vero cells that were treated with the combinations exhibited a different trend, with only Combination 2 reducing the levels of hMPV Phosphoprotein (P) gene (Figure 4D) and this correlates with Figure 3E, indicating the importance of naringenin for halting hMPV replication.

### 2.3. Increase of Intracellular Zinc Levels by the Natural Compound Combinations

**Figure 5:**
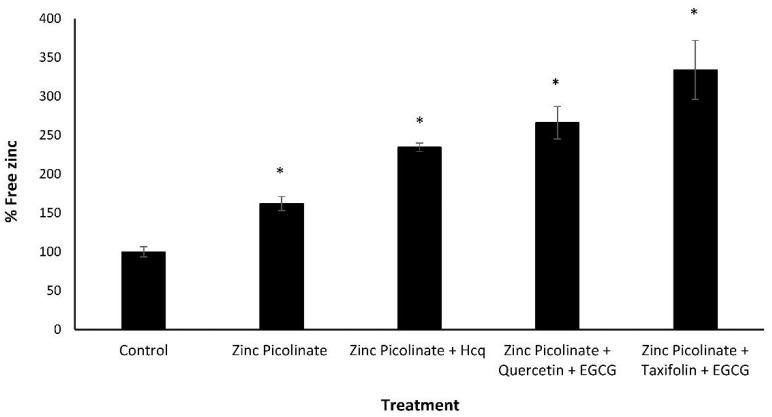
Free zinc quantification in treated A549 cells. A549 cells were treated with culture media containing the indicated combinations for four hours, after which the cells were trypsinized and lysed, and a free zinc quantification assay was performed. Free zinc levels are shown as percentages relative to the control (cells that were incubated with media containing solvents without compounds). Zinc picolinate, EGCG, quercetin, and taxifolin concentration is 40 µM. Hcq concentration is 20 µM. Values are means ± SD, Student’s *t*-test, * *p* < 0.05, all statistics are in relation to control.

The reduction in viral replication may result from an increased intracellular zinc concentration, leading to inhibition of intracellular virus proliferation. Accordingly, we quantified the effect of the different treatments on the intracellular levels of zinc. A549 cells were grown to 100% confluency and incubated for four hours on the following day with either one of several combinations of zinc and zinc ionophores. Then, the cells were trypsinized, lysed, and the free zinc in the lysates was quantified. Free zinc levels are shown as percentages relative to the control (cells incubated with medium containing the relevant solvents without the compounds). As can be seen in Figure 5, lysates of cells treated with the tested combinations had approximately three times higher free zinc levels compared to lysates of cells treated with zinc combined with Hcq (40 and 20 µM respectively) or with zinc alone (40 µM). Hcq concentration of above 20 µM had a cytotoxic effect on the cell line (data not shown). Thus, our results suggest a mechanistic explanation for the decrease in viral RNA replication: the combination of zinc (40 µM) with zinc ionophores (40 µM each) increases the intracellular levels of zinc, which in turn inhibits viral replication.

## 3. Materials and Methods

### 3.1. Materials

Reagents and Kits:

Zinc picolinate, Copper sulfate ≥ 99%, Epigallocatechin gallate (EGCG) ≥ 95%, Taxifolin ≥ 85%, Naringenin ≥ 95%, MTT reagent thiazolyl blue tetrazolium bromide (Sigma-Aldrich, Rehovot, Israel), Quercetin 95% (Angene, London, UK), Hydroxychloroquine sulfate 98% (Acros organics, Waltham, MA, USA), RNA purification Kit, EZ RNA, (Biological Industries, Beit HaEmek, Israel), cDNA kit, qScript Flex, (Quanta bio, Beverly, MA, USA), Fast SYBR Green Master Mix (Applied Biosystems, Waltham, MA, USA), Zinc quantification kit, and Fluorometric (Abcam, Cambridge, UK).

Cell lines:

The cell lines used in this study were A549 (ATCC^®^ CCL-185™), NCI-H1299 (ATCC^®^ CRL-5803™), Vero (ATCC^®^ CCL-81™), and SH-SY5Y (ATCC^®^ CRL-2266™).

Viral strains:

Influenza A virus (IAV) strain A/Puerto Rico/8/34 H1N1 (PR8) that harbors two copies of the mNeonGreen fluorescence marker, fused to the PB2 and PA polymerase ORFs, via a porcine teschovirus-1 2A peptide sequence, the human coronavirus OC43 (HCoV-OC43; a betacoronavirus) that was generously donated by Kobiler laboratory at Tel Aviv University, and human metapneumovirus expressing GFP (hMPV) [55].

### 3.2. Experimental Methods

#### 3.2.1. Cytotoxicity Evaluation Experiments

A549, H1299, and Vero cells (4 × 10^4^ cells/well) were cultured in DMEM/Nutrient Mixture F12 (Ham’s; 1:1), RPMI 1640, and DMEM high glucose Medium (Biological Industries), respectively, supplemented with 10% FBS in 96-well tissue microplates (100 µL per well) and allowed to adhere overnight at 37 °C. Only half of each plate was seeded with cells, with the other half later serving as a blank control containing medium alone. Specific compounds were dissolved in the following solvents: zinc picolinate in PBS (Hylabs, Rehovot, Israel), copper sulfate in UPW (Bio-Lab, Jerusalem, Israel), EGCG in cell growth media described above, quercetin, taxifolin, naringenin, and Hcq in DMSO (Acros organics, Waltham, MA, USA). Then, the dissolved compounds were added to the cell growth medium without FBS to attain a final volume of 1 mL for each treatment. Medium containing the equivalent volume of PBS, UPW, and DMSO served as a negative control, for which viability was normalized to 100%. For the combination treatments, the compounds were similarly dissolved in the relevant solvents at the following final concentrations: 40 µM zinc picolinate, 10 µM copper sulfate, 40 µM EGCG, 40 µM quercetin, 40 µM taxifolin, 40 µM naringenin, and 6 µM Hcq. The medium was replaced, and medium (100 µL) with or without the various compounds was added to each well. After 24 h incubation at 37 °C, cell viability was evaluated using 3-(4,5-dimethylthiazolyl-2)-2, 5-diphenyltetrazolium bromide MTT cell proliferation assay (Sigma-Aldrich) according to the manufacturer’s instructions. Briefly, after overnight incubation at 37 °C with the treatments, 10 µL of 5 mg/mL MTT reagent dissolved in PBS was added to each well, followed by additional four hours of incubation at 37 °C. Next, 100 µL DMSO was added to the wells, followed by 30 min incubation at 37 °C in the dark. Finally, color intensity was measured using a plate reader at 570 nm and background subtraction at 680 nm. The displayed results are representative of three biological experiments performed in triplicates. Values are means ± SD.

#### 3.2.2. Viral Infection and Quantification by FACS

A549 or Vero cells (1 × 10^5^ cells/300 µL/well) were cultured in a 24-well tissue microplate and allowed to adhere overnight at 37 °C. On the following day, cells were incubated with the control, Combination 1, Combination 2, or zinc/Hcq treatments (detailed above) for four hours, after which viral infection was performed. A total of 5 µL of GFP-labeled IAV (3 × 10^7^ PFU/mL, tittered on Madin-Darby canine kidney (MDCK) cells) were added to each well of A549 cells containing the treatments in medium without FBS. The cells were incubated in the presence of the virus for one hour, after which the virus-containing medium was replaced with a fresh medium containing only the indicated compositions without the virus. In the case of hMPV, 20 µL of the virus (6 × 10^6^ infectious units/mL tittered on Vero cells) were added to each well of Vero cells containing the treatments in medium with FBS. The cells were incubated in the presence of the virus for 48 h. Cells treated with the examined compositions but left uninfected served as a negative control. After the incubation, the cells were analyzed to quantify viral infection. The cells were trypsinized, washed, and fixed using 4% Paraformaldehyde in PBS. Then, the cells were sorted and analyzed by FACS. The percentage of GFP-positive cells represented the percentage of infected cells. The percentage values that were obtained for the infected and treated cells were normalized to the levels of the control (infected untreated) cells that were set as 100%. The results are representative of three biological repeats. Values are means ± SD, Student’s *t*-test, * *p* < 0.05, all statistics are in relation to control.

#### 3.2.3. Viral Infection and Quantification by qRT-PCR

H1299, A549, and Vero cells (1 × 10^5^ cells/300 µL/well) were cultured in a 24-well tissue microplate and allowed to adhere overnight at 37 °C. On the following day, cells were incubated with the control, Combination 1, Combination 2, or zinc/Hcq treatments (detailed above) for four hours, after which viral infection was performed. A total of 30 µL of HCoV-OC43 (2 × 10^5^ PFU/mL tittered on A549 cells) were added to each well of A549 and H1299 cells containing the treatments in medium with FBS for 24 h incubation. Regarding IAV and hMPV, the same infection protocol described above was performed. Next, viral replication quantification was done using qRT-PCR. For that purpose, the infected cells were trypsinized, and the detached cells were collected by centrifugation. RNA was then purified from the cells with EZ RNA purification kit (Biological Industries), according to the manufacturer’s protocol. Subsequently, the RNA was reverse transcribed using a commercial kit (Quanta bio) according to the manufacturer’s instructions. Finally, viral replication quantification levels were determined using qRT-PCR (StepOnePlus™ Real-Time PCR System, Applied Biosystems). 2 µL of the cDNA samples were mixed with 10 µL of Fast SYBR Green Master Mix, and 0.5 µM of both the forward and reverse viral gene primers (Table 1). A total of 6 µL of UPW were added to reach a final volume of 20 µL. The qRT-PCR optimized setup was 40 amplification cycles, with each cycle consisting of denaturation at 95 °C for 3 s and annealing at 60 °C for 30 s. A Relative Quantity (RQ) index of the viral genes was calculated based on the number of amplification cycles needed to exceed a required threshold. The housekeeping gene, GAPDH, was used as an endogenous reference for the PCR quantification. The RQ values were also normalized to the control infected untreated cells, set as “1”. The genes’ relative quantity represented the levels of viral infection in the cells. The results represent three biological repeats. Values are means ± SD, Student’s *t*-test, ** *p* < 0.01, *** *p* < 0.001, all statistics are in relation to control.

#### 3.2.4. Free Zinc Quantification Assay

A549 cells (1 × 10^5^ cells/500 µL/well) were cultured in 24-well tissue microplates and allowed to adhere overnight at 37 °C. On the following day, the cell medium was replaced with 500 µL medium without FBS, containing either of several combinations of zinc with polyphenolic zinc ionophores. Medium with the equivalent volume of PBS and DMSO was used as control. The cells were incubated with the various treatments for four hours, after which the free zinc concentration was measured using a fluorescence zinc quantification kit (Fluorometric, Abcam), according to the kit protocol. Briefly, cells were lysed with an EDTA-free lysis buffer and centrifuged to remove cell debris. Next, the cleared cell lysates were deproteinized by 7% TCA solution for five minutes and neutralized using 1 M Na_2_CO_3_ for five minutes on ice. ZnCl_2_ standards and the lysates of cells with the examined treatments were added to a 96-well black plate. Zinc assay buffer was added to each well and incubated for 10 min at room temperature, protected from light. Finally, the fluorescence signal was measured using a fluorescence plate reader at Excitation/Emission of 485/525 nm, respectively.

#### 3.2.5. qRT-PCR Primers

The following primers were used in this study (Table 1):

**Table 1:** The primers used in this study.

Virus/Cell Source	Primer Name	Sequence (5′-3′)
IAV	M2 Fw	CATGGAATGGCTAAAGACAAGACC
	M2 Rev	CCATTAAGGGCATTTTGGACA
hMPV	P Fw	GGCATGGGCAGACAACAGCGG
	P Rev	GAATTCCTCTTCTTCAACAGG
HCoV-OC43	HE Fw	GGTTGTGACTATATCGTACC
	HE Rev	GCGAATCAACAACCTGTACAG
A549 and H1299 cells	Human GAPDH Fw	AGCCACATCGCTCAGACAC
	Human GAPDH Rev	GCCCAATACGACCAAATCC
Vero cells	Monkey GAPDH Fw	GAAGGCTGGGGCTCATTTGC
	Monkey GAPDH Rev	ATGACGAACATGGGGGCGTC

## 4. Conclusions

Respiratory RNA viruses have been linked to a variety of illnesses. Influenza viruses and coronaviruses are two major respiratory viruses with pandemic potential [56,57]. COVID-19 is a worldwide public health emergency concern, and the increasing number of cases of this highly transmissible infection has highlighted the urgent need to find a satisfactory treatment [39]. As SARS-CoV-2 evolves rapidly, the currently available vaccines and antibody-based treatments would need to be updated frequently to match the circulating strains and avoid reinfection [21]. In an epidemic, therapeutics that inhibit the replication of viruses could be employed to treat severe disease and block the spread of infection [56]. This led us to develop active combinations of natural, GRAS dietary supplements that inhibit the replication of various RNA viruses, causing respiratory viral infections.

Zinc ions possess a wide range of antiviral activities [58], and thus, zinc supplementation at a proper dose has the potential to profoundly improve the elimination of both chronic and acute viral infections, as well as their pathologies and symptoms [31,34,53,59]. When zinc is combined with flavonoid zinc ionophores, the free intracellular zinc concentration increases. Flavonoids, in general, are central dietary supplements, and their notable antiviral activity against several viruses affecting humans may be of particular importance in the ongoing pandemic situation [42,45,46,47].

In this work, we established unique synergistic combinations based on zinc and polyphenolic flavonoid zinc ionophores to prevent COVID-19 (inferred by the infection of HCoV-OC43 [51]) and other RNA respiratory viral infections. Considering that we have shown that each of the compounds and their combinations are non-toxic and biocompatible in vitro, we deduce that they can be consumed for extended periods. Furthermore, we mimicked a scenario where such GRAS compounds are taken as prophylactics and demonstrated that the combinations are highly effective against various RNA viruses in vitro. Importantly, as the compounds are known for their diverse antiviral activities [32,60,61], we infer that viral inhibition by the combinations examined is rather general and thus should be helpful against a variety of viruses, particularly RNA viruses associated with winter respiratory infections.

Moreover, the advantage of combining different polyphenols is that the mixture helps protect against a variety of RNA viruses as each compound has an affinity to different organs, tissues, and cell types. To conclude, the SARS-CoV-2 pandemic imposes unmet medical needs. Here, we suggest a method to attenuate the replication of this pathogen and other respiratory RNA viruses in a way that likely will not be affected by future mutations and new variants of SARS-CoV-2. This method may also be synergistic with other antiviral drugs and vaccines. The value of these combinations awaits *in vivo* and human trials.

## Data Availability

Data is contained within the article and supplementary material.

## Supplementary Materials

The following are available online at https://www.mdpi.com/article/10.3390/ph15030377/s1, Figure S1: Toxicity evaluation of the examined compounds and combinations in neuroblastoma cells by MTT, Figure S2: Fluorescence microscope images of A549 cells infected with mNeonGreen–labeled IAV.
