# Supplementary material for: Inhibition of Respiratory RNA Viruses by a Composition of Ionophoric Polyphenols with Metal Ions
Source: Pharmaceuticals (Basel). 2022 Mar 20;15(3):377. doi: 10.3390/ph15030377 (PMC8955458; doi:10.3390/ph15030377)
Supplement: Supplementary file 1 [file pharmaceuticals-15-00377-s001.zip › pharmaceuticals-1621691-supplementary.pdf]

Supplementary Information

Supplementary results

This file includes:

- Supplementary Figure S1. Toxicity evaluation of the examined compounds and Combinations in neuroblastoma cells by MTT.
- Supplementary Figure S2. Fluorescence microscope images of A549 cells infected with mNeonGreen–labeled IAV.

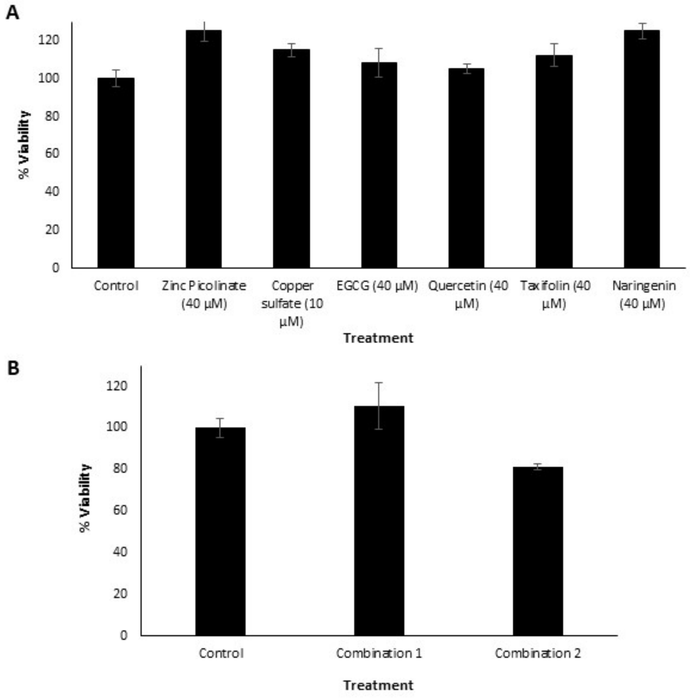

Figure S1: Toxicity evaluation of the examined compounds and Combinations in neuroblastoma cells by MTT. **A**. SH-SY5Y neuroblastoma cells were treated with the indicated compounds and concentrations. **B**. SH-SY5Y cells treated with culture media containing either of the two dietary supplements Combinations: Combination 1 or Combination 2. Cells, incubated with medium containing the relevant solvents without the compounds, served as the control. The cells were incubated with the Combinations for 24 hours, followed by the addition of the MTT reagent. Absorbance was determined at 570 nm and 680 nm. The results represent three biological repeats; values are means ± SD.

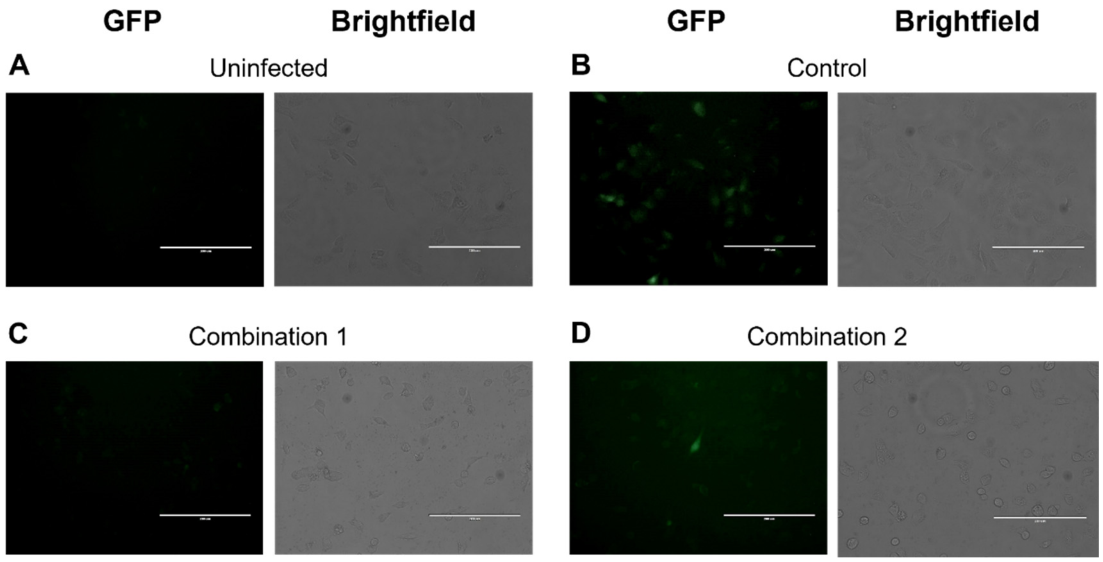

Figure S2: Fluorescence microscope images of A549 cells infected with mNeonGreen–labeled IAV. **A**. Uninfected cells. **B–D**. Fluorescence and brightfield images of A549 cells that were infected with IAV virus and treated with media containing **B**. the solvents in which the compounds were dissolved (control), **C**. Combination 1 or **D**. Combination 2. Scale bars: 200 µm.
